# Bio-Based Self-Healing Epoxy Vitrimers with Dynamic Imine and Disulfide Bonds Derived from Vanillin, Cystamine, and Dimer Diamine

**DOI:** 10.3390/molecules29204839

**Published:** 2024-10-12

**Authors:** Itsuki Abe, Mitsuhiro Shibata

**Affiliations:** Department of Applied Chemistry, Faculty of Engineering, Chiba Institute of Technology, 2-17-1, Tsudanuma, Narashino 275-0016, Chiba, Japan; s20a6006np@s.chibakoudai.jp

**Keywords:** bio-based vitrimer, dynamic covalent bond, imine and disulfide bonds, vanillin, cystamine, dimer diamine, healing properties, thermal and mechanical properties

## Abstract

The condensation reactions of 4,4′-(ethane-1,2-diylbis (oxy)) bis(3-methoxybenzaldehyde) (VV) with cystamine, 1,6-hexamenthylene diamine, and a dimer diamine (Priamine^TM^ 1075) produced three types of vanillin-derived imine-and disulfide-containing diamines (VC, VH, and VD, respectively). Thermal curing reactions of polyglycerol polyglycidyl ether with VD and mixtures of VC/VD and VH/VD produced bio-based epoxy vitrimers (BEV-VD, BEV-VC/VD, and BEV-VH/VD, respectively). The degree of swelling and gel fraction tests revealed the formation of a network structure, and the crosslinking density increased with a decreasing VD fraction. The glass transition temperature, tensile strength, and tensile modulus of the cured films increased as the VD fraction decreased. In contrast, the thermal degradation temperature of the cured films increased as the VD fraction increased. All the cured films were healed by hot pressing at 120 °C for 2 h under 1 MPa at least three times. The healing efficiencies, based on tensile strength after the first healing treatment, were 75–78%, which gradually decreased as the healing cycle was repeated. When imine-and disulfide-containing BEV-VC/VD and imine-containing BEV-VH/VD with the same VC/VD and VH/VD ratios were used, the former exhibited a slightly higher healing efficiency.

## 1. Introduction

Bio-based vitrimers are polymer networks derived from renewable resources containing exchangeable dynamic covalent bonds (e.g., esters, imines, boronic acid esters, and disulfide bonds). They exhibit self-healing properties and material recyclability, which cannot be achieved using conventional thermoset polymers, mitigating the consumption of petroleum resources and enabling the achievement of carbon neutrality [1,2,3,4,5,6,7,8]. Vanillin (VN) is a promising compound for the preparation of bio-based vitrimers because it is currently one of the only industrially available bio-based formyl phenols [9]. Its aldehyde group can be readily converted into a dynamic imine bond through a reaction with primary amines, and its phenolic hydroxy group can be readily converted into various functional groups [10,11]. Active research has recently focused on VN-based vitrimers containing dynamic imine bonds [10]. The majority of these studies deal with VN-based vitrimers derived from epoxy resins, which constitute some of the most representative thermosetting resins [12,13,14,15,16,17,18,19,20,21,22,23,24,25,26,27,28,29,30,31]. There is also a limited range of papers that discuss the derivation of VN-based vitrimers from bio-based epoxy resins. Liu et al. reported reprocessable VN-based epoxy vitrimers prepared by curing epoxidized soybean oil (ESO) with an imine-containing aminophenol (IAP) derived from VN and *p*-aminophenol [15]. Roig et al. reported a reprocessable VN-based epoxy vitrimer prepared by curing a mixture of trimethylolpropane triglycidyl ether and an imine-containing diglycidyl ether derived from VN, 4,4′-oxydianiline, and epichlorohydrin with poly(oxypropylene) diamines [21]. Wang et al. prepared reprocessable VN-based epoxy vitrimers by curing an imine-containing diglycidyl ether derived from VN, *p*-phenylenediamine, and epichlorohydrin with cystamine (CTA) [24]. Monteserin et al. reported reprocessable VN-based epoxy vitrimers prepared by curing epoxidized linseed oil with an imine-containing bisphenol derived from VN and 4,4′-diaminodiphenylmethane [25]. Zhou et al. reported a reprocessable and flame-retardant VN-based epoxy vitrimer prepared by curing a diglycidyl ether derived from glycerol triglycidyl ether (GTE) and 9,10-dihydro-9-oxa-10-phosphaphenanthrene-10-oxide using IAP [27]. Guggari et al. prepared a VN-based epoxy vitrimer by curing an imine-containing diglycidyl ether derived from VN, epichlorohydrin, and 1,6-hexamenthylene diamine (HMDA) with CTA [28]. Verdugo et al. prepared reprocessable VN-based epoxy vitrimers by curing imine-and disulfide-containing diglycidyl ether derived from VN and CTA with isophorone diamine, *m*-xylylene diamine, 1,2-diaminecyclohexane, or tris(2-aminoethyl)amine [31]. Although these studies included examinations of the reprocessability of the produced material, they did not include analyses of the self-healing properties. Zhao et al. reported a VN-based epoxy vitrimer prepared by curing an imine-containing diglycidyl ether derived from VN, *p*-aminophenol, and epichlorohydrin with a polyetheramine (Jeffamine). The vitrimer was weldable by treating at 100 °C and 10 N for 60 s and then 120 °C for 4 h [12]. Zhao et al. reported VN-based epoxy vitrimers prepared by curing ESO with an imine-containing bisphenol derived from VN and 4,4′-diaminodiphenylmethane [17]. The vitrimers were reprocessable by treating their small cut pieces at 190 °C under 20 MPa for 5 min and weldable by treating at 150 °C for 20 min. Zhao et al. reported VN-based epoxy vitrimers prepared by curing GTE with methylhexahydrophthalic anhydride (MHHPA) and an imine-containing bisphenol (VT) derived from VN and tyamine [30]. The vitrimers were reprocessable by hot pressing their small cut pieces at 190 °C, for 1 h, and weldable by pressing and treating at 160 °C for 2 h. Although these studies investigated the weldability, i.e., healability of the overlapped two cut pieces of a cured film by hot pressing, the self-healability of the two cut pieces contacted with the cut surface and repetitive self-healing properties were not studied in detail. Furthermore, the biomass contents of these materials were not so high, because the bio-based reagents were partially used.

**Scheme 1:**
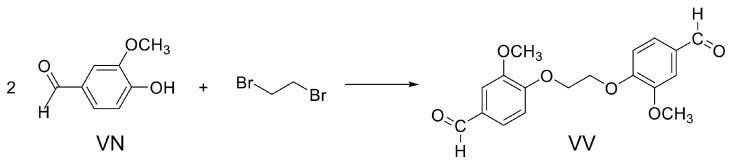
Synthesis of VV by the reaction of VN and 1,2-dibromoethane.

**Scheme 2:**
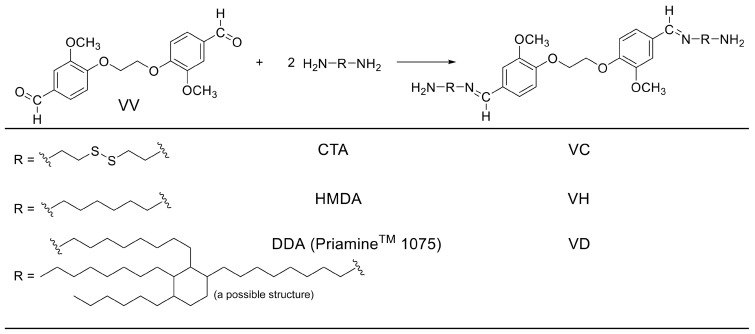
Synthesis of VC, VH, and VD by the reactions of VV with CTA, HMDA, and DDA.

**Scheme 3:**
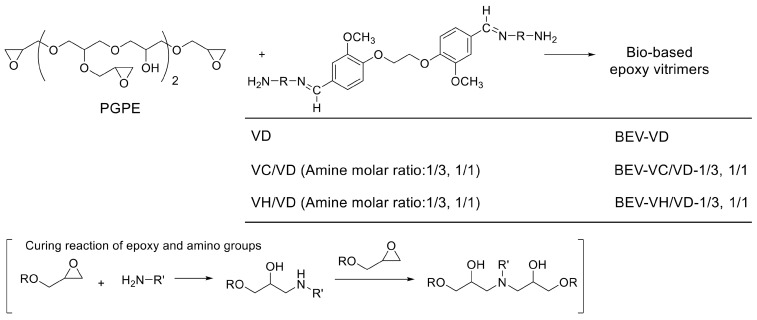
Preparation of bio-based epoxy vitrimers by the thermal curing reactions of PGPE with VD and mixtures of VC/VD and VH/VD.

In this study, the condensation reactions of a VN dimer (VV) derived from VN and 1,2-dibromoethane (Scheme 1) with CTA, HMDA, and a dimer diamine (DDA, Priamine^TM^ 1075) produced VC with imine and disulfide bonds, VH with only imine bonds, and VD with imine bonds and flexible aliphatic chains, respectively (Scheme 2). The thermal curing reactions of polyglycerol polyglycidyl ether (PGPE), a bio-based glycerol-derived epoxy resin with a functionality of approximately 4 with VD and mixtures of VC/VD and VH/VD produced bio-based epoxy vitrimers (BEV-VD, BEV-VC/VD, and BEV-VH/VD, respectively) (Scheme 3). The thermal and mechanical properties and repetitive self-healing properties driven by the exchange reactions of dynamic imine and disulfide bonds in the materials were investigated in detail. Because VN, CTA, and DDA are derived from renewable resources among the reagents used for epoxy hardeners, the biomass contents (98.4–98.7 wt%) of the cured films are very high when the epichlorohydrin used during the preparation of PGPE is obtained from glycerol [32,33].

## 2. Results and Discussion

### 2.1. Preparation and Characterization of the BEV-VC/VD and BEV-VH/VD Films

**Figure 1:**
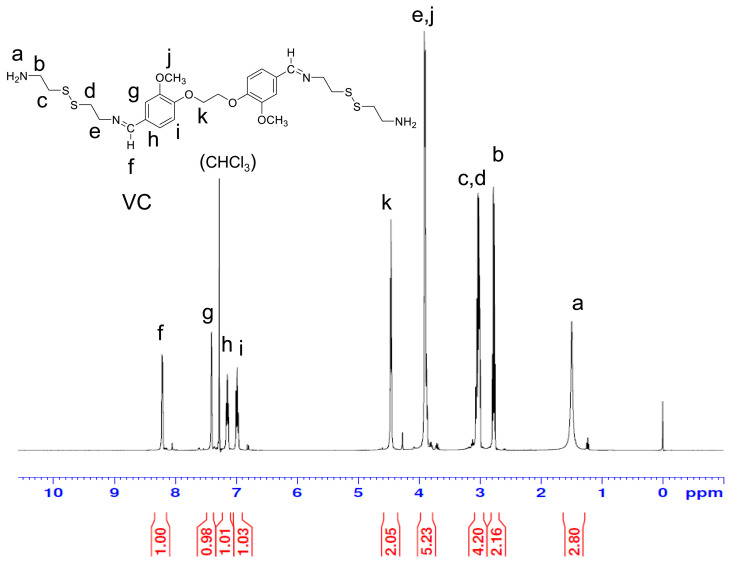
The 400 MHz ^1^H-NMR spectrum of VC in CDCl_3_ at 300 K. Red numeral values represent integral values of respective chemical shift regions.

**Figure 2:**
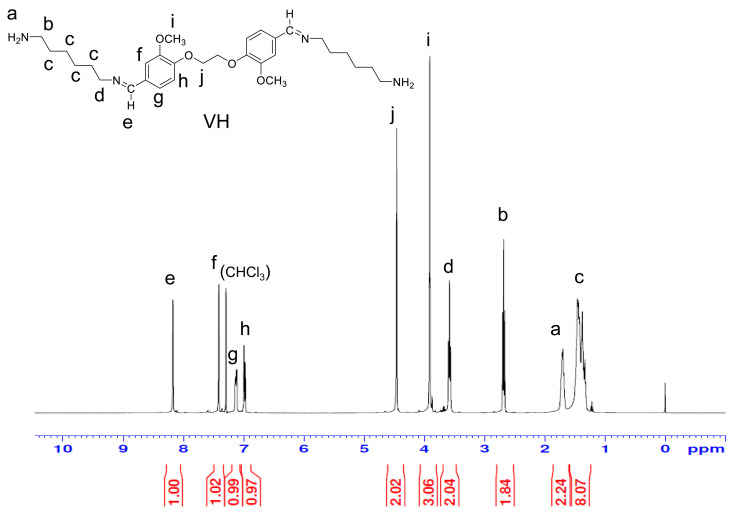
The 400 MHz ^1^H-NMR spectrum of VH in CDCl_3_ at300 K. Red numeral values represent integral values of respective chemical shift regions.

**Figure 3:**
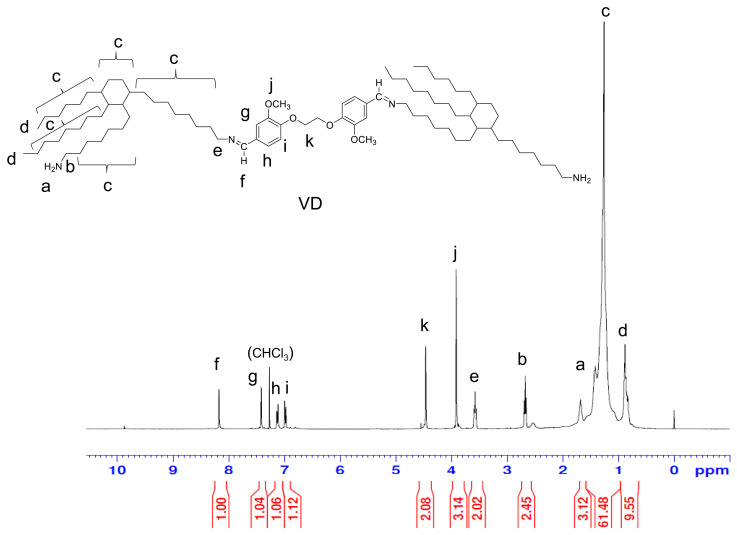
The 400 MHz ^1^H-NMR spectrum of VD in CDCl_3_ at 300 K. Red numeral values represent integral values of respective chemical shift regions.

The reactions of VV with CTA, HMDA, and DDA at a CHO/NH_2_ ratio of 1/2 produced imine-containing diamines (VC, VH, and VD, respectively) (Scheme 2). Figure 1, Figure 2 and Figure 3 show the ^1^H-NMR spectra of VC, VH, and VD, respectively, in CDCl_3_. In their ^1^H-NMR spectra, an imine ^1^H-signal was observed in a relatively low magnetic field region of δ 8.1–8.3 ppm (s), and no aldehyde ^1^H-signal, which was observed at 9.8 ppm for VV (Figure S1, Supplementary Material), was detected, indicating that the aldehyde groups of VV reacted completely with primary amino groups of CTA, HMDA, and DDA. The ^1^H-signals of terminal amine-substituted methylene (H-b) and dioxyethylene (H-k for VC and VD; H-j for VH) units of VC, VH, and VD were observed in an alkyl region of δ 2.6–2.8 ppm (m or t) and 4.4–4.5 ppm (s), respectively. The integral ratios (2.16/2.05, 1.84/2.02, and 2.45/2.08 = 1.05/1, 0.91/1, and 1.18/1 for VC, VH, and VD, respectively) suggest that CTA–VV–CTA, HMDA–VV–HMDA, and DDA–VV–DDA diamines were primarily produced, considering that the theoretical integral ratio became 1/1.

**Figure 4:**
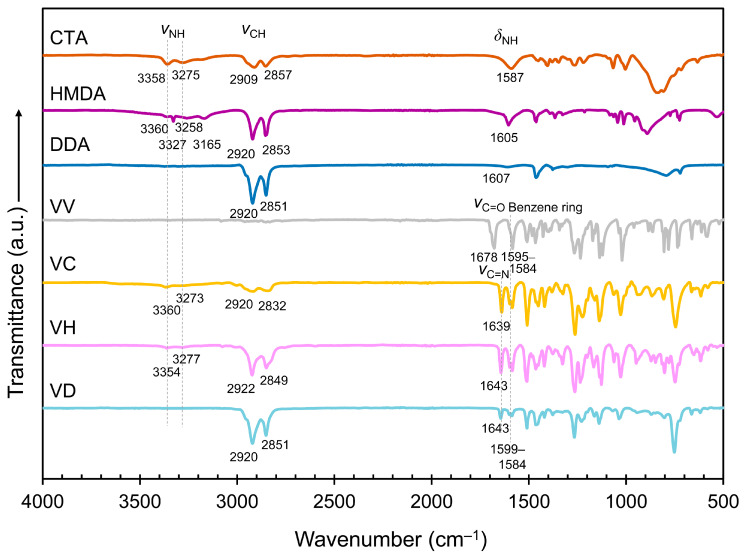
FT-IR spectra of CTA, HMDA, DDA, VV, VC, VH, and VD.

Figure 4 shows the FT-IR spectra of VC, VH, VD, and their reactants. In the FT-IR spectra of VC, VH, and VD, an absorption band caused by the imine C=N stretching vibration (*ν*_C=N_) and overlapping bands attributed to the primary amine scissors (δ_NH_2__) and benzene framework vibrations were observed at 1639–1643 cm^−1^ and 1584–1599 cm^−1^, respectively. In addition, an absorption band corresponding to the aldehyde C=O stretching vibration (*ν*_C=O_), which was observed at 1678 cm^−1^ for VV, was not detected, which is in accordance with the above-mentioned ^1^H-NMR result. Absorption bands corresponding to the primary amine asymmetric and symmetric NH (*ν*_NH_) vibrations, which were observed for CTA and HMDA, were also observed at 3360, 3273 cm^−1^, and 3354, 3277 cm^−1^ for VC and VH, respectively. However, *ν*_NH_ bands were not clearly detected for DDA and VD, which is probably because the content of the primary amino groups was minimal. The ^1^H-NMR and FT-IR spectral data confirmed that VC, VH, and VD had the desired chemical structures. In the next epoxy curing reaction step, the reaction solutions of VC, VH, and VD in chloroform were used directly without isolating the diamines.

**Figure 5:**
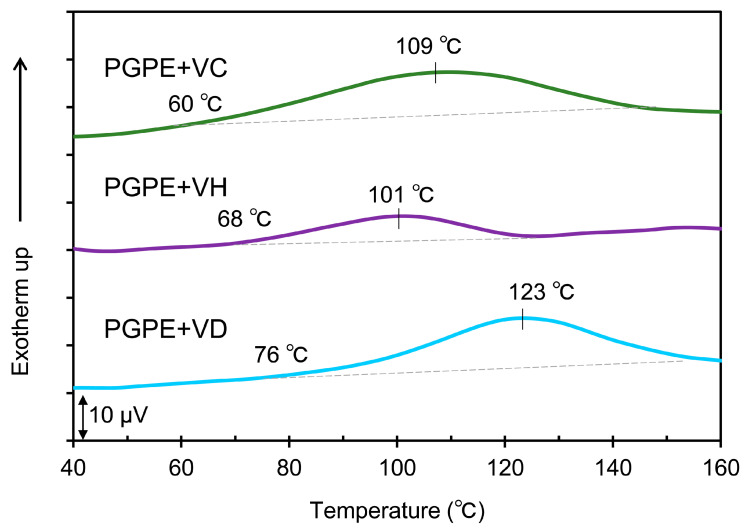
DTA curves of mixtures of PGPE/VC, PEGE/VH, and PGPE/VD at an epoxy/NH_2_ molar ratio of 2/1.

Before thermally curing the PGPE with VC, VH, and VD, DTA measurements of the PGPE/VC, PGPE/VH, and PGPE/VD mixtures at an epoxy/NH_2_ ratio of 2/1 were carried out to determine the curing temperature. The onset and peak temperatures of the broad exothermic peaks attributed to the epoxy/amine reactions of the PGPE/VC, PGPE/VH, and PGPE/VD mixtures were observed at ca. 60 and 109 °C, 68 and 101 °C, and 76 and 123 °C, respectively (Figure 5), indicating that the amino groups of VD exhibit a lower reactivity toward the epoxy groups of PGPE than those of CTA and HMDA. It is considered that the reaction of terminal amino groups of VD with epoxy groups is sterically hindered by the presence of DDA-based dialkylcyclohexane rings. Based on the DTA results, the drying and curing temperatures of PGPE/VC/VD and PGPE/VH/VD solutions in chloroform were set to 50 °C and 80–130 °C, respectively. The thermal curing reactions of PGPE and VD, PGPE and mixtures of VC/VD with amine molar ratios of 1/3 and 1/1, and PGPE and mixtures of VH/VD with amine molar ratios of 1/3 and 1/1 at an epoxy/NH_2_ molar ratio of 2/1 produced BEV-VD, BEV-VC/VD-1/3, and 1/1, and BEV-VH/VD-1/3 and 1/1 films, respectively (Scheme 3). Solutions of VC, VH, and VD in chloroform, which were synthesized in situ, were used to prepare the cured films. All the cured films were obtained as brown flat films (Figure S2, Supplementary Material).

**Figure 6:**
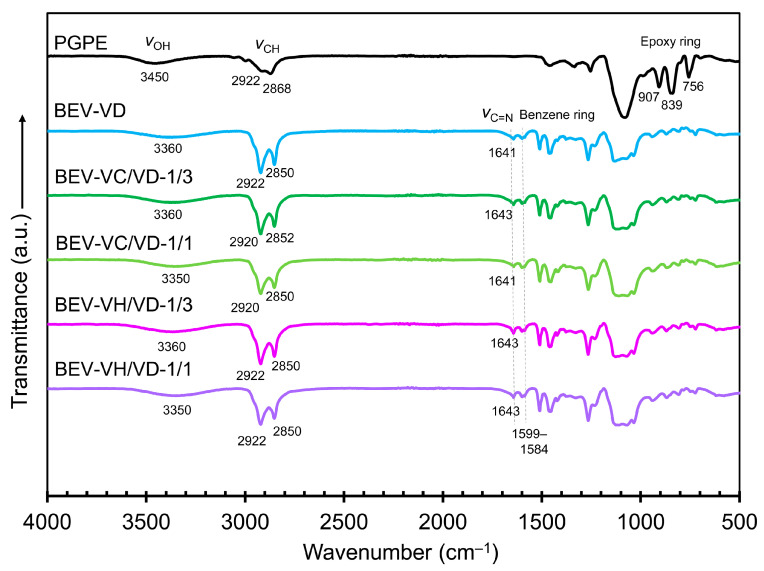
FT-IR spectra of PGPE and BEV-VD, BEV-VC/VD, and BEV-VH/VD films.

Figure 6 shows the FT-IR spectra of the PGPE and cured films. In the FT-IR spectra of the cured films, absorption peaks characteristic of epoxy groups, which were observed at 756, 839, and 907 cm^−1^ for PGPE, were not detected, and a broad absorption band attributed to the OH stretching vibration (*ν*_OH_), imine *ν*_C=N_ band, and benzene framework bands were observed at approximately 3350–3360 cm^−1^, 1641–1643 cm^−1^, and 1584–1599 cm^−1^, respectively. These results suggested that imine-containing epoxy-cured products were produced by epoxy-amine curing reactions.

**Figure 7:**
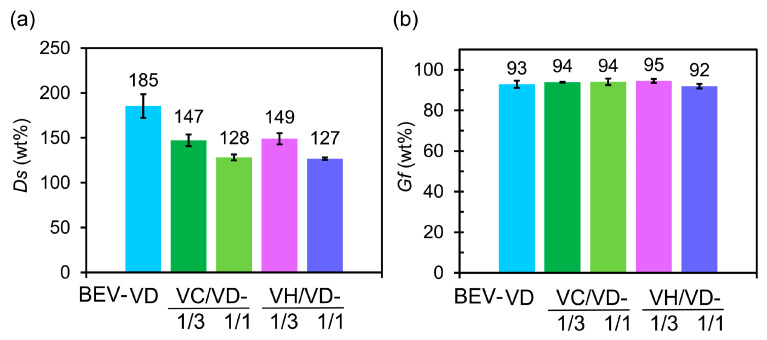
(**a**) *Ds* and (**b**) *Gf* values of the cured films.

Figure 7 shows the *Gf* and *Ds* values of the cured products, which were measured using chloroform as the extraction and soaking solvent. The *Gf* values of all the cured products were higher than 90 wt%, indicating the formation of epoxy networks. The *Ds* values decreased with decreasing VD fractions, indicating that the crosslinking density increased in the same order. This result should be caused by the following factors: the distance between terminal two amino groups of VD is much longer than those of VC and VH; the decrease in VD fractions causes the increase in crosslinking density of the cured product; and the increase in crosslinking density causes the decrease in hollow parts into which chloroform can infiltrate. This finding can be attributed to the fact that the distances between the terminal amino groups of CTA and HMDA were shorter than those of DDA.

### 2.2. Thermal Properties of the BEV-VD, BEV-VC/VD, and BEV-VH/VD Films

**Figure 8:**
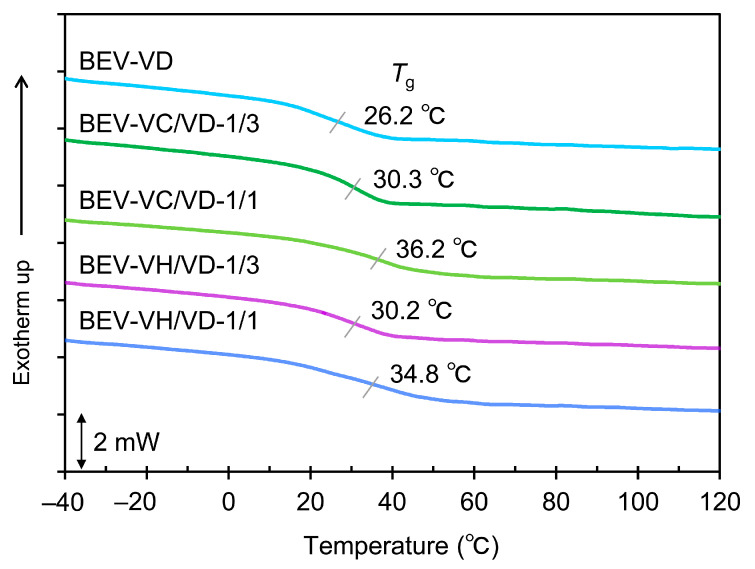
DSC curves of the cured films.

Figure 8 shows the DSC curves of the cured films. The *T*_g_s of the cured films increased with decreasing VD fractions. This is because the mobility of molecular chains is suppressed by the increase in crosslinking density. When cured films with the same VD fraction were compared, the *T*_g_s of BEV-VC/VD-1/3 and 1/1 (30.3 and 36.2 °C) were found to be comparable to those of BEV-VH/VD-1/3 and 1/1 (30.2 and 34.8 °C), respectively, reflecting that the distance between terminal amino groups of VC is comparable to that of VH. The *T*_g_s of the cured products in this study were higher than those (15–24 °C) of the cured products (GMVs) of GTE, MHHPA, and VT [30].

**Figure 9:**
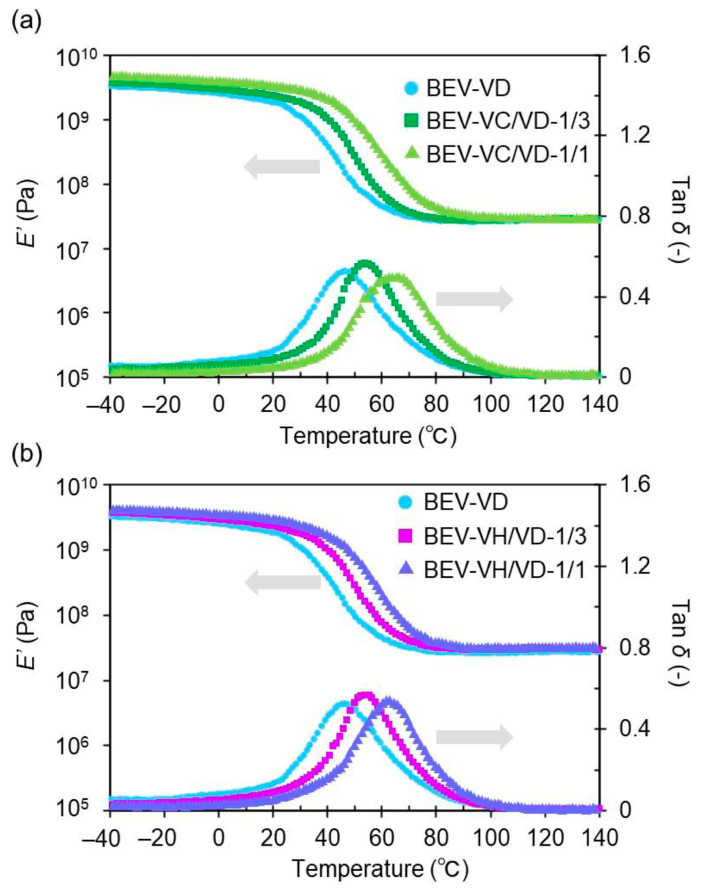
DMA curves of (**a**) BEV-VD, BEV-VC/VD-1/3, and BEV-VC/VD-1/1, and (**b**) BEV-VD, BEV-VH/VD-1/3, and BEV-VH/VD-1/1. The DMA curves indicated by left and right arrows are values of *E*′ and tan *δ*, respectively.

Figure 9 shows the DMA curves of the cured films. Table 1 summarizes *T*_α_, *E*′ at 20 °C, *E*′ at (*T*_α_ + 50) °C, and *v*_e_ values. The *T*_α_ and *v*_e_ values of the cured films increased with a decreasing VD fraction based on the results of DSC and swelling tests. The *E*′ at 20 °C of the cured films also increased with a decreasing VD fraction, indicating that DDA possessed longer flexible aliphatic chains than CTA and HMDA.

**Figure 10:**
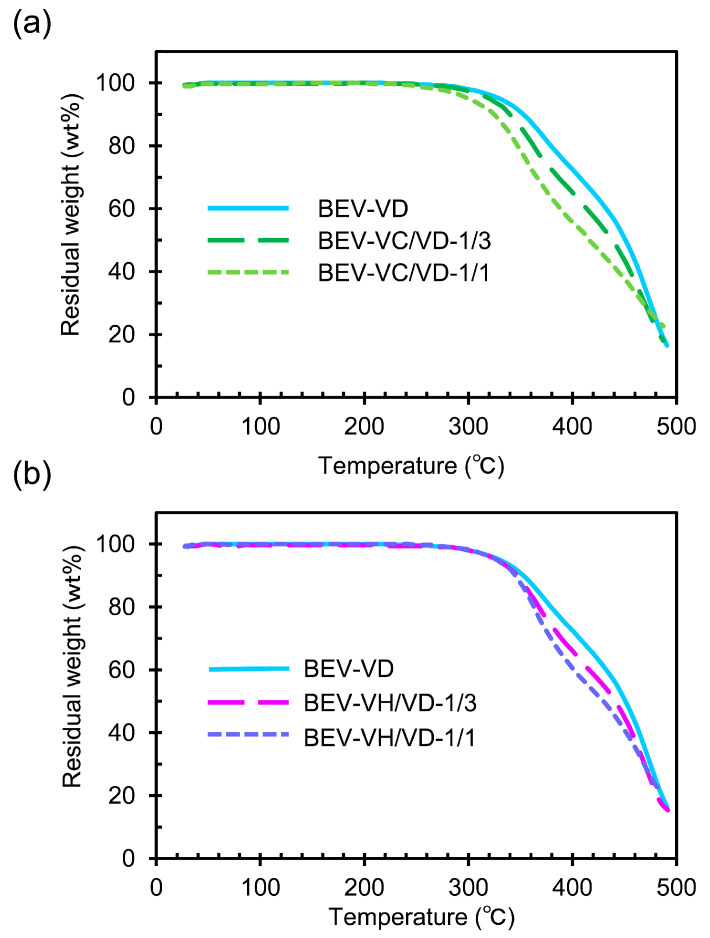
TGA curves of (**a**) BEV-VD, BEV-VC/VD-1/3, and BEV-VC/VD-1/1, and (**b**) BEV-VD, BEV-VH/VD-1/3, and BEV-VH/VD-1/1.

Figure 10 shows the TGA curves of the cured films. The TGA curves of VC, VH, and VD are shown in Figure S3 (Supplementary Material). Table 2 summarizes the *T*_d5%_, *T*_d10%_, and *T*_d50%_ values of the cured VC, VH, and VD films. All the cured films exhibited *T*_d5%_ values higher than 300 °C. The *T*_d5%_, *T*_d10%_, and *T*_d50%_ values of the cured films decreased with decreasing VD fractions, indicating that the *T*_d5%_, *T*_d10%_, and *T*_d50%_ values of VC and VH were substantially lower than those of VD. When the cured films with the same VD fraction were compared, the *T*_d5%_, *T*_d10%_, and *T*_d50%_ values of BEV-VC/VD-1/3 and 1/1 were lower than those of BEV-VH/VD-1/3 and 1/1, respectively, indicating that the *T*_d10%_ and *T*_d50%_ values of VC were lower than those of VH. The *T*_d5%_ values (303–330 °C) of the cured products in this study were higher than those (203–281 °C) of GMVs [30].

### 2.3. Mechanical and Self-Healing Properties of the BEV-VD, BEV-VC/VD, and BE-VH/VD Films

**Figure 11:**
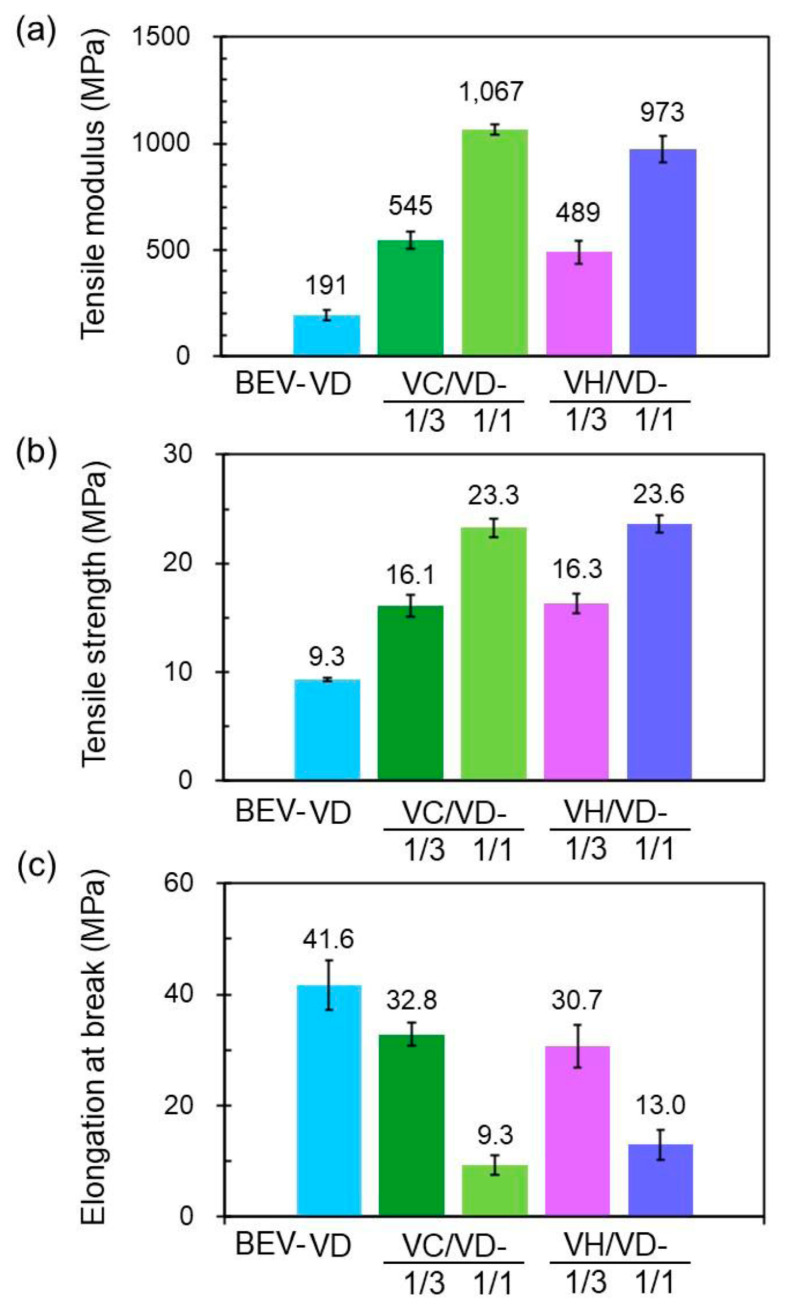
(**a**) Tensile strength, (**b**) tensile modulus, and (**c**) elongation at break of the cured films.

The mechanical properties of the cured films were evaluated using tensile testing. The stress–strain curves of the cured films are shown in Figure S4 (Supplementary Material). Figure 11 shows the tensile modulus and strength and the elongation at break of the cured films. The tensile modulus and strength of the cured films increased with a decreasing VD fraction, and the elongation at break decreased in the same order owing to an increase in crosslinking density, resulting in an increase in stiffness of the materials. When the cured films with the same VD fraction were compared, the tensile strength and modulus and the elongation at break of BEV-VC/VD-1/3 and 1/1 were comparable to those of BEV-VH/VD-1/3 and 1/1, respectively. The tensile strengths (16–24 MPa) and elongations at break (9–33%) of BEV-VC/VD and BEV-VH/VD films were comparable to those (16–21 MPa and 9–11%, respectively) of GMVs [30]. Also, the tensile strengths (16–24 MPa) and tensile moduli (489–1067 MPa) of BEV-VC/VD and BEV-VH/VD films were much higher than those (0.5 MPa and 4.7 MPa, respectively) of a cured product of commercially available PGPE and polyetheramine (Jeffamine ED-600) [34].

**Figure 12:**
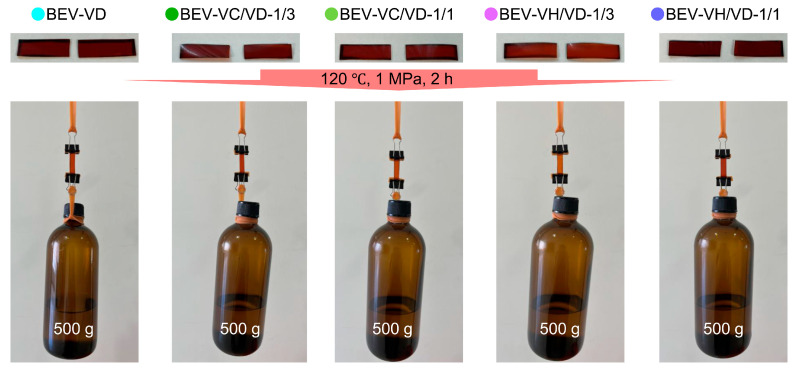
Healing behavior of the cured film by hot pressing at 120 °C under 1 MPa for 2 h.

**Figure 13:**
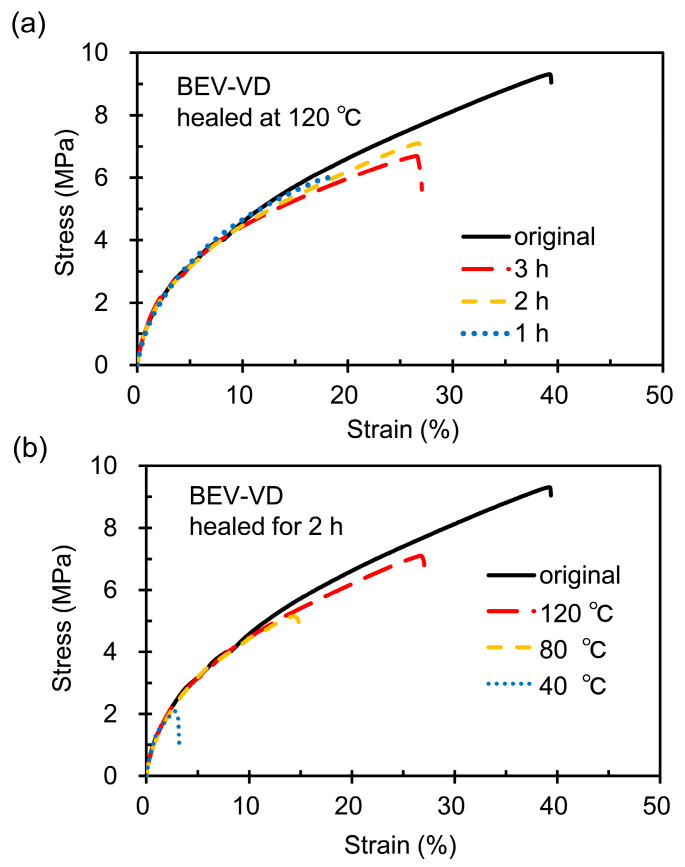
Change in the tensile stress–strain curves of BEV-VD films (**a**) healed at 120 °C under 1 MPa for 1, 2, and 3 h, and (**b**) healed at 40, 80, and 120 °C under 1 MPa for 2 h.

The as-prepared BEV-VD film was cut into two pieces. The pieces were then contacted with the cut surface at 120 °C for 15 min and then pressed at 120 °C under 1 MPa for 2 h, adhering them together to form a healed (h1) film (Figure 12). All the other cured films were healed using the same method. None of the healed samples broke even under a loading of 500 g. Figure 13 shows the influence of healing time and temperature on the tensile stress–strain curves of the BEV-VD films before and after healing at 120 °C under 1 MPa for 1, 2, and 3 h and before and after healing at 40, 80, and 120 °C under 1 MPa for 2 h. When the healing temperature was 120 °C, the tensile strength and strain at break of the film healed for 2 h were higher than those for the film healed for 1 h, and they were comparable to those for the film healed at 3 h. When the healing time was 2 h, the tensile strength and strain at break of the healed samples increased with increasing healing temperature in the temperature range of 40–120 °C. Considering that the curing temperature of the original BEV-VD was 120 °C, the healing conditions of all the cured films were set to 120 °C under 1 MPa for 2 h.

**Figure 14:**
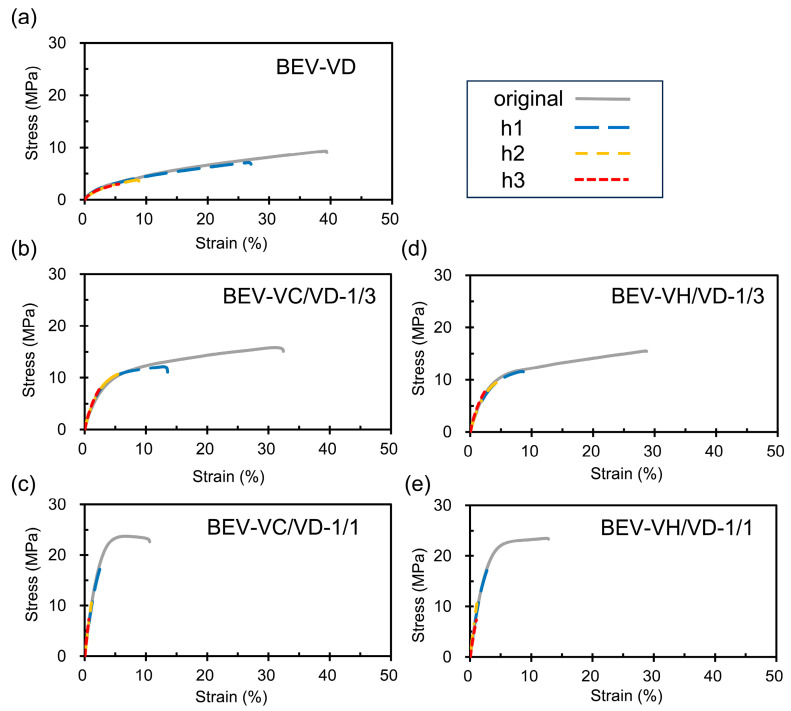
Tensile stress–strain curves of (**a**) BEV-VD, (**b**) BEV-VC/VD-1/3, (**c**) BEV-VC/VD-1/1, (**d**) BEV-VH/VD-1/3, and (**e**) BEV-VH/VD-1/1, healed at 120 °C under 1 MPa for 2 h once, twice, and three times.

All the cured films were healed at least three times by hot pressing at 120 °C under 1 MPa for 2 h. The FT-IR spectra of h1-, h2-, and h3-BEV-VD, BEV-VC/VD, and BEV-VH/VD films are shown in Figure S5 (Supplementary Materials). No evident change in the FT-IR spectra of the h1-, h2-, and h3-films was observed, indicating that side reactions did not occur during the exchange of imine and disulfide bonds. Figure 14 shows the changes in the tensile stress–strain curves of the cured films that were subjected to repetitive healing. The initial slope (i.e., tensile modulus) of the curves remained almost unchanged after repetitive healing. However, the maximum stress and strain decreased with an increase in the number of healing treatments. Table 3 summarizes the *η*_σ_ values of h1-, h2-, and h3-films and imine and disulfide contents of the cured films. The tensile moduli, tensile strengths, and elongations at break of the original, h1-, h2-, and h3-films are also summarized in Table S1 (Supplementary Material). The *η*_σ_ values of the h1-, h2-, and h3-BEV-VC/VD-1/3 with the imine and disulfide contents of 2.9 and 1.7 wt% and h1-, h2-, and h3-BEV-VH/VD-1/3 with the imine content of 2.9 wt% were higher than those of h1-, h2-, and h3-BEV-VD with the imine content of 2.6 wt%, respectively, reflecting an increase in dynamic covalent bonds. However, the *η*_σ_ values of h2-and h3-BEV-VC/VD-1/1 with the imine and disulfide content of 3.2 and 3.8 wt% were lower than those of h2-and h3-BEV-VC/VD-1/3, respectively, even though the content of dynamic covalent bonds for BEV-VC/VD-1/1 was higher than that for BEV-VC/VD-1/3. In addition, the *η*_σ_ values of h2-and h3-BEV-VH/VD-1/1 with the imine content of 3.3 wt% were lower than those of h2-and h3-BEV-VH/VD-1/3, respectively. These results were attributed to an increase in the stiffness of the cured films with increasing VC/VD and VH/VD ratios, leading to the inhibition of the exchange reaction of dynamic covalent bonds. However, the *η*_σ_ values of h1-BEV-VC/VD-1/1 and h1-BEV-VH/VD-1/1 were comparable to those of h1-BEV-VC/VD-1/3 and h1-BEV-VH/VD-1/3, respectively, and the former exhibited much a higher tensile strength and modulus than the latter. When the h1-, h2-, and h3-BEV-VC/VD and h1-, h2-, and h3-BEV-VH/VD with the same VD content were compared, the former exhibited slightly higher *η*_σ_ values. This result can be attributed to the presence of dynamic imine and disulfide bonds in the former, whereas the latter contained only dynamic imine bonds.

## 3. Materials and Methods

### 3.1. Materials

PGPE (trade name: DENACOL^®^ EX-512, with an epoxy equivalent weight of 167 g eq.^−1^, average functional group number of 4, chlorine content of 6.5%, and viscosity of 1300 mPa s at 25 °C) was supplied by Nagase ChemteX. Corp. (Tokyo, Japan). VN, 1,2-dibromoethane, and CTA dihydrochloride (purity ≥ 97%) were purchased from Tokyo Chemical Industry (Tokyo, Japan). HMDA (purity ≥ 98%) was purchased from Kanto Chemical Co. Inc. (Tokyo, Japan). DDA (Priamine^TM^ 1075, with an amine equivalent weight of 272.4 g eq.^−1^) was supplied by Cargill Japan LLC (Tokyo, Japan). CTA (colorless viscous liquid) was prepared by the reaction between CTA dihydrochloride and sodium hydroxide, as previously described [35]. All commercially available reagents were used as received without further purification.

### 3.2. Synthesis of VV

VV was synthesized according to a previously reported method [36]. A typical procedure is as follows: a mixture of 1,2-dibromoethane (18.79 g, 100.0 mmol), VN (30.43 g, 200.0 mmol), sodium hydroxide (8.80 g, 220 mmol), and potassium iodide (1.66 g, 10 mmol) was refluxed at 100 °C in water (300 mL) for 24 h under vigorous stirring in a nitrogen atmosphere. The product was isolated via vacuum filtration and suspended in water (300 mL). After vacuum filtration, the wet cake was resuspended in water/ethanol (*v*/*v*:1/1, 200 mL). This procedure was performed in ethanol (200 mL). After vacuum filtration, the wet cake was vacuum-dried at 100 °C for 24 h to produce VV as a white powder (18.2 g) with a yield of 55%. ^1^H NMR (DMSO-*d*_6_) *δ* (ppm) 3.83 (s, 6H), 4.48 (s, 4H), 7.27 (d, 2H), 7.42 (sl, 2H), 7.57 (dd, 2H), and 9.86 (s, 2H).

### 3.3. Synthesis of VC, VH, and VD

A solution of VV (0.520 g, 3.15 mmol-CHO) and CTA (0.480 g, 6.30 mmol-NH_2_) in chloroform (20 mL) was stirred at 60 °C for 1 h. The resulting solution was evaporated under reduced pressure at 70–80 °C and then dried in vacuo at 100 °C for 1 h to produce VC as a viscous pale-yellow oil with a quantitative yield. Similarly, VH and VD were synthesized using VV (0.587 g, 3.55 mmol-CHO) and HMDA (0.413 g, 7.11 mmol-NH_2_), and VV (0.233 g, 1.41 mmol-CHO) and DDA (0.767 g, 2.82 mmol-NH_2_), respectively. VH and VD were obtained as a white solid and viscous pale-yellow oil, respectively. VC: ^1^H-NMR (CDCl_3_), *δ* (ppm) 1.49 (s, 5.6H, H-a), 2.77 (m, 4.3H, H-b), 3.02 (m, 8.4H, H-c,d), 3.90 (m, 8.4H, H-e,j), 4.46 (s, 4.1H, H-k), 6.98 (m, 2.0H, H-i), 7.14 (m, 2.0H, H-h), 7.40 (sl, 2.0H, H-g), 8.21 (s, 2.0H, H-f). VH: ^1^H-NMR (CDCl_3_), *δ* (ppm) 1.3–1.6 (m, 16.1H, H-c), 1.70 (sl, 4.5H, H-a), 2.68 (t, 3.7H, H-b, *J*_bc_-13.8 Hz), 3.58 (t, 4.1H, H-d, *J*_dc_-13.8 Hz), 3.90 (s, 6.1H, H-i), 4.45 (s, 4.0H, H-j), 6.98 (dl, 1.9H, H-h, *J*_hg_-8.3 Hz), 7.12 (dl, 2.0H, H-g, *J*_gh_-8.3 Hz), 7.41 (sl, 2.0H, H-fs), 8.17 (s, 2.0H, H-e). VD: ^1^H-NMR (CDCl_3_), *δ* (ppm) 0.88 (m, 19.1H, H-d), 1.1–1.6 (m, 123.0H, H-c), 1.68 (s, 6.2H, H-a), 2.67 (t, 4.9H, H-b, *J*_bc_-13.8 Hz),), 3.57 (t, 4.0H, H-e, *J*_ec_-13.8 Hz), 3.91 (s, 6.3H, H-j), 4.46 (s, 4.2H, H-k), 6.98 (dl, 2.2H, H-i, *J*_ih_-8.2 Hz), 7.12 (dl, 2.1H, H-h, *J*_hi_-8.2 Hz), 7.41 (sl, 2.1H, H-g), 8.17 (s, 2.0H, H-f).

### 3.4. Preparation of BEV-VD, BEV-VC/VD, and BEV-VH/VD Films

Solutions of VV (0.262 g, 1.58 mmol-CHO) and CTA (0.241 g, 3.17 mmol-NH_2_) in chloroform (20 mL) and VV (0.785 g, 4.75 mmol-CHO) and DDA (2.59 g, 9.51 mmol-NH_2_) in chloroform (20 mL) were stirred at 60 °C for 1 h to produce solutions of VC and VD in chloroform, respectively. The feed CHO/NH_2_ ratio was 1/2. To the mixed solutions of VC and VD, a solution of PGPE (2.12 g, 12.67 mmol-epoxy) in chloroform (20 mL) was added, and this was stirred at room temperature for 30 min. The resulting solution was poured into a culture dish (internal diameter: 10 mm) containing perfluoroalkoxy alkanes and dried at room temperature for 24 h. The obtained film was dried at 50 °C for 24 h, 80 °C for 2 h, 100 °C for 2 h, and 130 °C for 20 h in an electric oven to produce a BEV-VC/VD-1/3 film (thickness 0.8 mm). The feed epoxy/NH_2_ ratio was 2/1. BEV-VD, BEV-VC/VD-1/1, and BEV-VH/HD-1/3, 1/1 were prepared by following a procedure similar to that used to prepare BEV-VC/VD-1/3. The amounts of feed reactants for all the cured products are listed in Table 4.

### 3.5. Self-Healing Analysis Experiments

Rectangular samples (45 × 7 × (0.6–0.8) mm^3^) of the as-prepared BEV-VC/VD or BEV-VH/VD films were cut into halves. The two pieces were contacted with the cut surface at approximately 120 °C for 15 min, sandwiched between two Teflon sheets and stainless steel plates, and hot-pressed at 120 °C under 1 MPa for 2 h using Mini Test Press-10 to obtain healed BEV-VC/VD or BEV-VH/VD (h1-BEV-VC/VD or h1-BEV-VH/VD) films. This healing cycle was repeated three times (h1, h2, and h3). The healing efficiency was evaluated by calculating the tensile strength recovery rate using the following equation:

*η*_σ_ (%) = 100*σ*_1_/*σ*_0_ (1)

where *σ*_0_ and *σ*_1_ are the average tensile strengths of the original and healed samples, respectively.

### 3.6. Measurements

Proton nuclear magnetic resonance (^1^H-NMR) spectra were recorded with a Bruker Ascend 400 MHz spectrometer (Madison, WI, USA) using CDCl_3_ or DMSO-d6 as the solvent. Differential thermal analysis (DTA) was performed using a Shimadzu DTA-50 instrument (Shimadzu Corp., Kyoto, Japan) at a heating rate of 10 °C min^−1^ in a nitrogen atmosphere. Fourier-transform infrared (FT-IR) spectra were recorded on a Shimadzu IRAffinity-1S instrument (Shimadzu Corp., Kyoto, Japan) in the 4000–500 cm^−1^ range using the attenuated total reflectance method. The FT-IR spectra were acquired using 50 scans at a resolution of 4 cm^−1^. To quantify the degree of swelling (*Ds*), the film (10 × 10 × (0.6–0.8) mm^3^) was soaked in chloroform at room temperature for 24 h. The following equation was used:

*Ds* (%) = 100(*w*_1_ − *w*_0_)/*w*_0_ (2)

where *w*_0_ is the initial weight of the film and *w*_1_ is the weight of the swollen film after soaking. The film dipped in chloroform was dried at 130 °C in an electric oven for 6 h. The gel fraction (*Gf*) obtained by chloroform extraction was calculated using the following equation:

*Gf* (%) = 100 *w*_2_/*w*_0_ (3)

where *w*_0_ and *w*_2_ represent the weights of the original and dried films, respectively. Three samples of each film were tested, and the mean values and standard deviations were calculated from the degree of swelling and gel fraction measurements. Differential scanning calorimetry (DSC) was performed using a Shimadzu DSC-60Plus instrument (Shimadzu Corp., Kyoto, Japan) in a nitrogen atmosphere. After the as-prepared sample (8–9 mg) was cooled to −50 °C, the heating scan was monitored at a heating rate of 20 °C min^−1^. The glass transition temperature (*T*_g_) was determined from the midpoint of the heat flow change. Dynamic mechanical analysis (DMA) (DMA1, Mettler–Toledo, Japan) on a rectangular plate sample (10 × 5 × (0.6–0.8) mm^3^) was performed with a chuck distance of 10 mm, frequency of 1 Hz, and heating rate of 10 °C min^−1^. The amplitude for the DMA measurements was 10 μm. The loss tangent (tan δ) peak temperature (*T*_α_) was obtained from the temperature dependency of tan δ. The crosslinking densities (*v*_e_) of the cured products were calculated using the following equation:

*v*_e_ = *E*′/(3*RT*) (4)

where *R* is the gas constant (8.314 J mol K^−1^), *E*′ is the storage modulus of the cured film in the rubbery region at (*T*_α_ + 50) K, and *T* is the absolute temperature at which the storage modulus values are obtained [37]. Thermogravimetric analysis (TGA) was performed on a sample weighing approximately 3–5 mg using a Shimadzu TGA-50 thermogravimetric analyzer (Shimadzu Corp., Kyoto, Japan) at a heating rate of 20 °C min^−1^ in a nitrogen atmosphere. The temperatures at which *x*% mass loss occurred (*T*_d*x*%_, *x* = 5, 10, and 50) were determined using the TGA curves. The tensile tests of the rectangular samples (45 × 7 × (0.6–0.8) mm^3^) were performed at a temperature of 20–25 °C, using a Shimadzu Autograph AG-I instrument (Shimadzu Corp., Kyoto, Japan). The span length and testing speed were 25 and 6 mm min^−1^, respectively. Five specimens were tested for each set of samples, and the mean values and standard deviations were calculated.

## 4. Conclusions

The condensation reactions of VV with CTA, HMDA, and DDA (Priamine^TM^ 1075, Cargill Incorp., Minnetonka, MN, USA) produced VC with imine and disulfide bonds, VH with imine bonds, and VD with imine bonds and flexible long aliphatic chains. The thermal curing reactions of PGPE with VD and mixtures of VC/VD and VH/VD produced bio-based epoxy vitrimers (BEV-VD, BEV-VC/VD, and BEV-VH/VD, respectively). PGPE, VV, CTA, and DDA are bio-based reagents. The degree of swelling, gel fraction, and DMA analyses revealed the formation of a network structure, and the crosslinking density increased with a decreasing VD fraction. The *T*_g_, *T*_α_, tensile strength, and tensile modulus of the cured films increased with a decreasing VD fraction. Conversely, the *T*_d5%_, *T*_d10%_, and *T*_d50%_ values of the cured films increased with an increasing VD fraction. All the cured films were healed via hot pressing at 120 °C for 2 h under 1 MPa at least three times. The *η*_σ_ values for the first healing treatment were 75–78% and gradually decreased as the healing cycle was repeated. When the imine-and disulfide-containing BEV-VC/VD and imine-containing BEV-VH/VD with the same VD fraction were compared, the former exhibited a slightly higher healing efficiency. Because the bio-based epoxy vitrimers in this study have high biomass content, excellent self-healing properties, and good thermal and mechanical properties, they are expected to be applied to fields such as environmentally benign coatings and electronic components.

## Figures and Tables

**Table 1:** *T*_α_, *E*′ at 20 °C, *E*′ at (*T*_α_ + 50) °C, and *v*_e_ values measured by DMA for the cured films.

Sample	*T*_α_ (°C)	*E*′ (MPa) at 20 °C	*E*′ (MPa) at (*T*_α_ + 50) °C	*v*_e_ (mmol cm^–3^)
BEV-VD	46.5	1875	26.43	2.87
BEV-VC/VD-1/3	53.8	2350	27.60	2.94
BEV-VC/VD-1/1	65.3	3361	30.11	3.11
BEV-VH/VD-1/3	55.2	2351	31.09	3.30
BEV-VH/VD-1/1	61.6	3026	32.22	3.36

**Table 2:** *T*_d5%_, *T*_d10%_, and *T*_d50%_ values of the cured VC, VH, and VD films.

Sample	*T*_d5%_ (°C)	*T*_d10%_ (°C)	*T*_d50%_ (°C)
BEV-VD	330	353	450
BEV-VC/VD-1/3	321	341	438
BEV-VC/VD-1/1	303	325	416
BEV-VH/VD-1/3	327	346	440
BEV-VH/VD-1/1	327	345	429
VC	147	179	359
VH	131	206	398
VD	337	376	462

**Table 3:** The change in η_σ_ values caused by repetitive healing (h1, h2, and h3) and imine and disulfide content of the cured films.

Sample	*η*_σ_ (%) of h1-Sample	*η*_σ_ (%) of h2-Sample	*η*_σ_ (%) of h3-Sample	Imine Content (wt%)	Disulfide Content (wt%)
BEV-VD	74.7 ± 8.9	39.4 ± 4.1	32.8 ± 1.3	2.6	-
BEV-VC/VD-1/3	77.8 ± 5.0	61.2 ± 7.5	48.4 ± 3.1	2.9	1.7
BEV-VC/VD-1/1	76.8 ± 3.9	46.3 ± 3.3	33.6 ± 4.5	3.2	3.8
BEV-VH/VD-1/3	75.5 ± 6.7	56.0 ± 4.8	41.4 ± 2.7	2.9	-
BEV-VH/VD-1/1	76.3 ± 6.7	46.3 ± 4.8	31.6 ± 1.5	3.3	-

**Table 4:** The amounts of feed reactants of the cured products.

Sample	VC *^1^		VH *^1^		VD *^1^		PGPE *^2^ g (mmol-epoxy)
	VV g (mmol-CHO)	CTA g (mmol-NH_2_)	VV g (mmol-CHO)	HMDA g (mmol-NH_2_)	VV g (mmol-CHO)	DDA g (mmol-NH_2_)	
BEV-VD	0.948 (5.74)	-		-		3.13 (11.48)	1.92 (11.48)
BEV-VC/VD-1/3	0.262 (1.58)	0.241 (3.17)		-	0.785 (4.75)	2.59 (9.51)	2.12 (12.67)
BEV-VC/VD-1/1	0.584 (3.53)	0.538 (7.07)		-	0.584 (3.53)	1.93 (7.07)	2.37 (14.14)
BEV-VH/HD-1/3	-	-	0.264 (1.60)	0.186 (3.20)	0.793 (4.80)	2.61 (9.60)	2.14 (12.80)
BEV-VH/VD-1/1	-	-	0.597 (3.61)	0.420 (7.22)	0.597 (3.61)	1.97 (7.22)	2.42 (14.45)

*^1^ The CHO/NH_2_ molar ratio was 1/2 for the synthesis of VC, VH, and VD. *^2^ The epoxy/NH_2_ molar ratio was 2/1 for the preparation of the bio-based epoxy vitrimers.

## Data Availability

All data are available in the manuscript.

## Supplementary Materials

The following supporting information can be downloaded at https://www.mdpi.com/article/10.3390/molecules29204839/s1, Figure S1. ^1^H-NMR spectrum of VV in DMSO-*d*_6_. Figure S2. Appearance of the cured films. Figure S3. TGA curves of VC, VH, and VD. Figure S4. Tensile stress–strain curves of the cured films. Figure S5. FT-IR spectra of h1-, h2-, and h3-BEV-VC, BEV-VC/VD, and BEV-VH/VD films. Table S1. Tensile moduli, tensile strengths and elongation at breaks of original-, h1-, h2-, and h3-BEV-VD, BEV-VC/VD, and BEV-VH/VD films.
