# Supplementary material for: Bio-Based Self-Healing Epoxy Vitrimers with Dynamic Imine and Disulfide Bonds Derived from Vanillin, Cystamine, and Dimer Diamine
Source: Molecules. 2024 Oct 12;29(20):4839. doi: 10.3390/molecules29204839 (PMC11510012; doi:10.3390/molecules29204839)
Supplement: Supplementary file 1 [file molecules-29-04839-s001.zip › molecules-3243533-supplementary.pdf]

Supplementary Material

Diamine Bio-Based Self-Healing Epoxy Vitrimers with Dynamic Imine and Disulfide Bonds Derived from Vanillin, Cystamine, and Dimer Diamine

Itsuki Abe and Mitsuhiro Shibata *

Department of Applied Chemistry, Faculty of Engineering, Chiba Institute of Technology, 2-17-1, Tsu-danuma, Narashino 275-0016, Chiba, Japan; s20a6006np@s.chibakoudai.jp

* Correspondence: mitsuhiro.shibata@p.chibakoudai.jp

Contents

Figure S1. The 400 MHz ^{1}H-NMR spectrum of VV in DMSO-d_6 at 300 K. Red numeral values represent integral values of respective chemical shift regions.

Figure S2. Appearance of the cured films.

Figure S3. TGA curves of VC, VH, and VD.

Figure S4. Tensile stress-strain curves of (a) BEV-VC/VD-1/1, BEV-VC/VD-1/3, and BEV-VD, and (b) BEV-VH/VD-1/1, BEV-VH/VD-1/3, and BEV-VD.

Figure S5. FT-IR spectra of h1-, h2-, and h3-BEV-VD, BEV-VC/VD, and BEV-VH/VD films.

Table S1. Tensile moduli, tensile strengths and elongation at breaks of original-, h1-, h2-, and h3-BEV-VD, BEV-VC/VD, and BEV-VH/VD films.

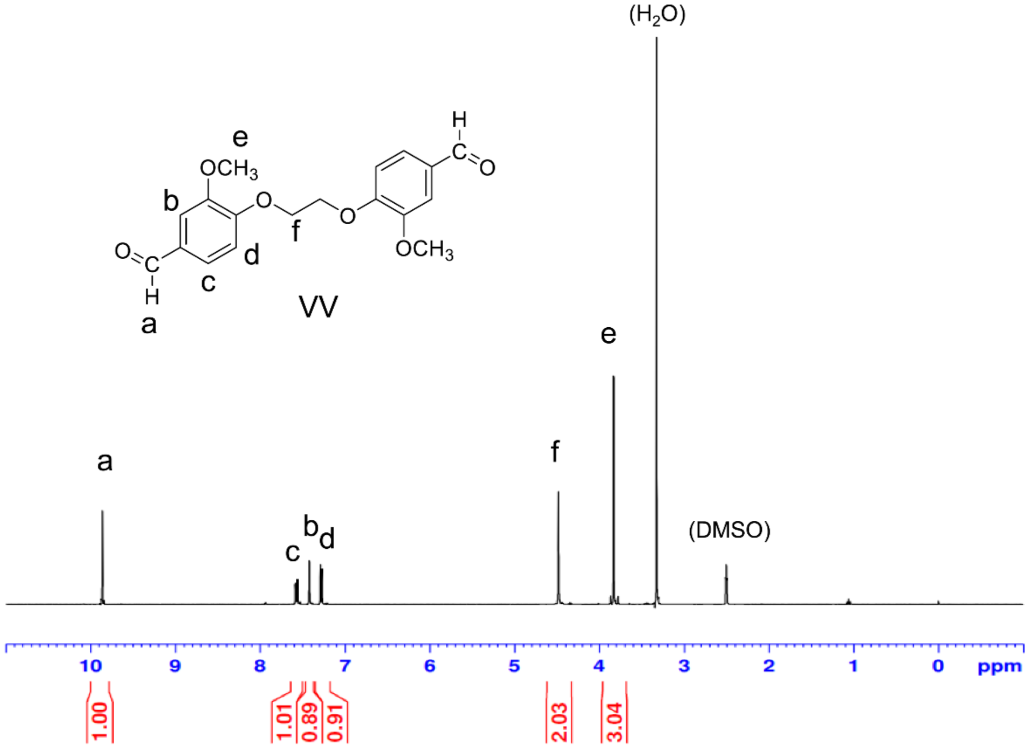

Figure S1. The 400 MHz ^{1}H-NMR spectrum of VV in DMSO-d_6 at 300 K. Red numeral values represent integral values of respective chemical shift regions.

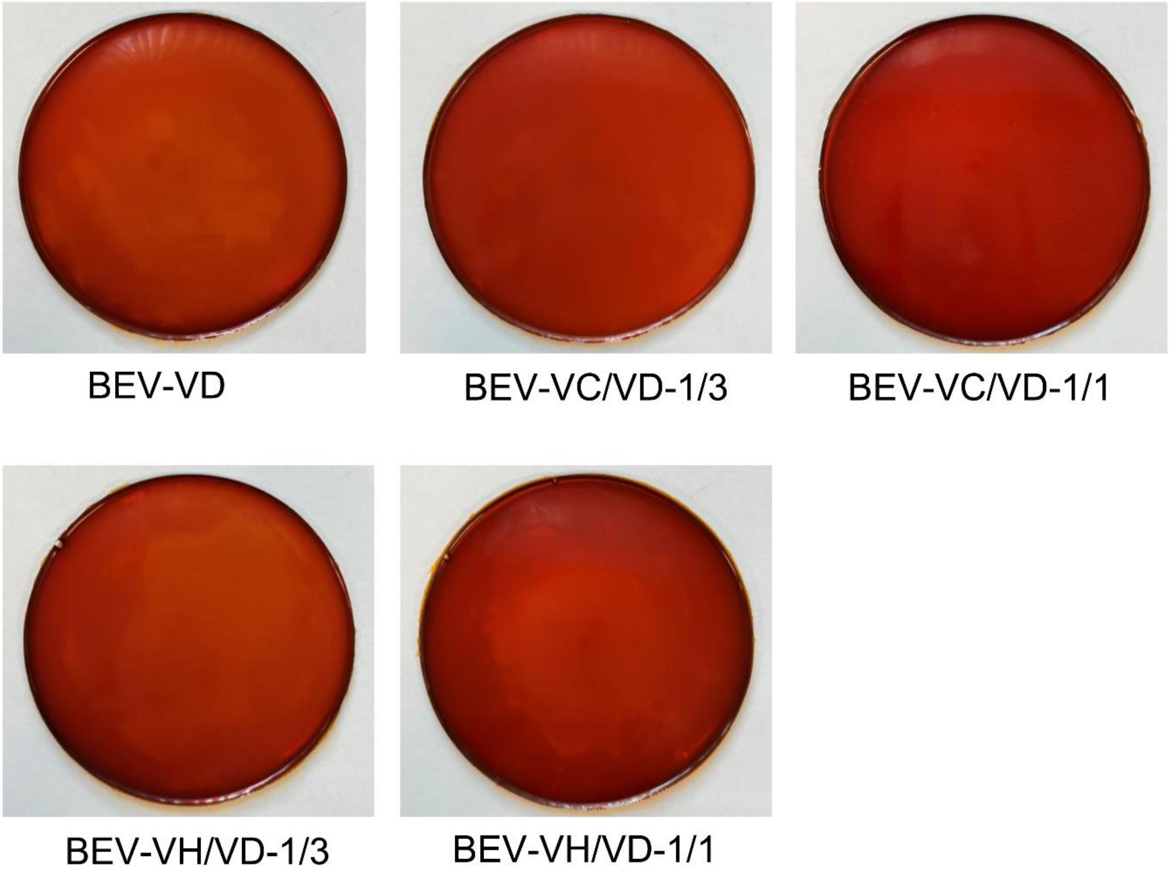

Figure S2. Appearance of the cured films.

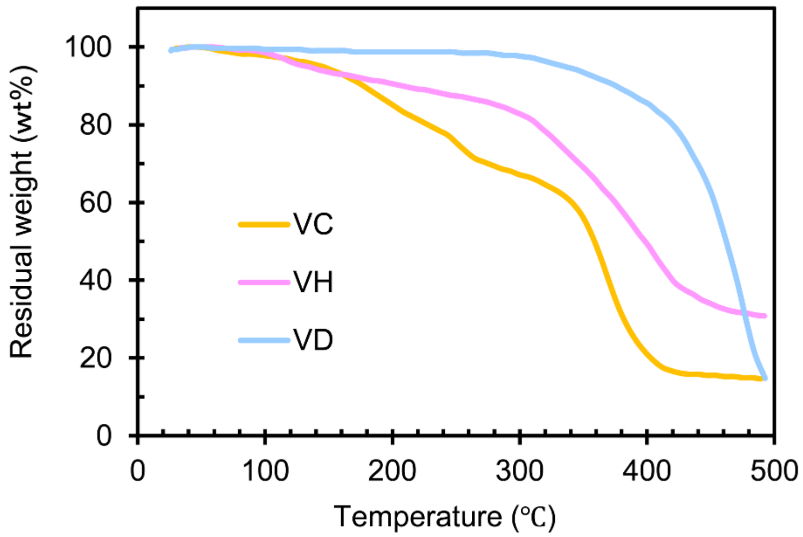

Figure S3. TGA curves of VC, VH, and VD.

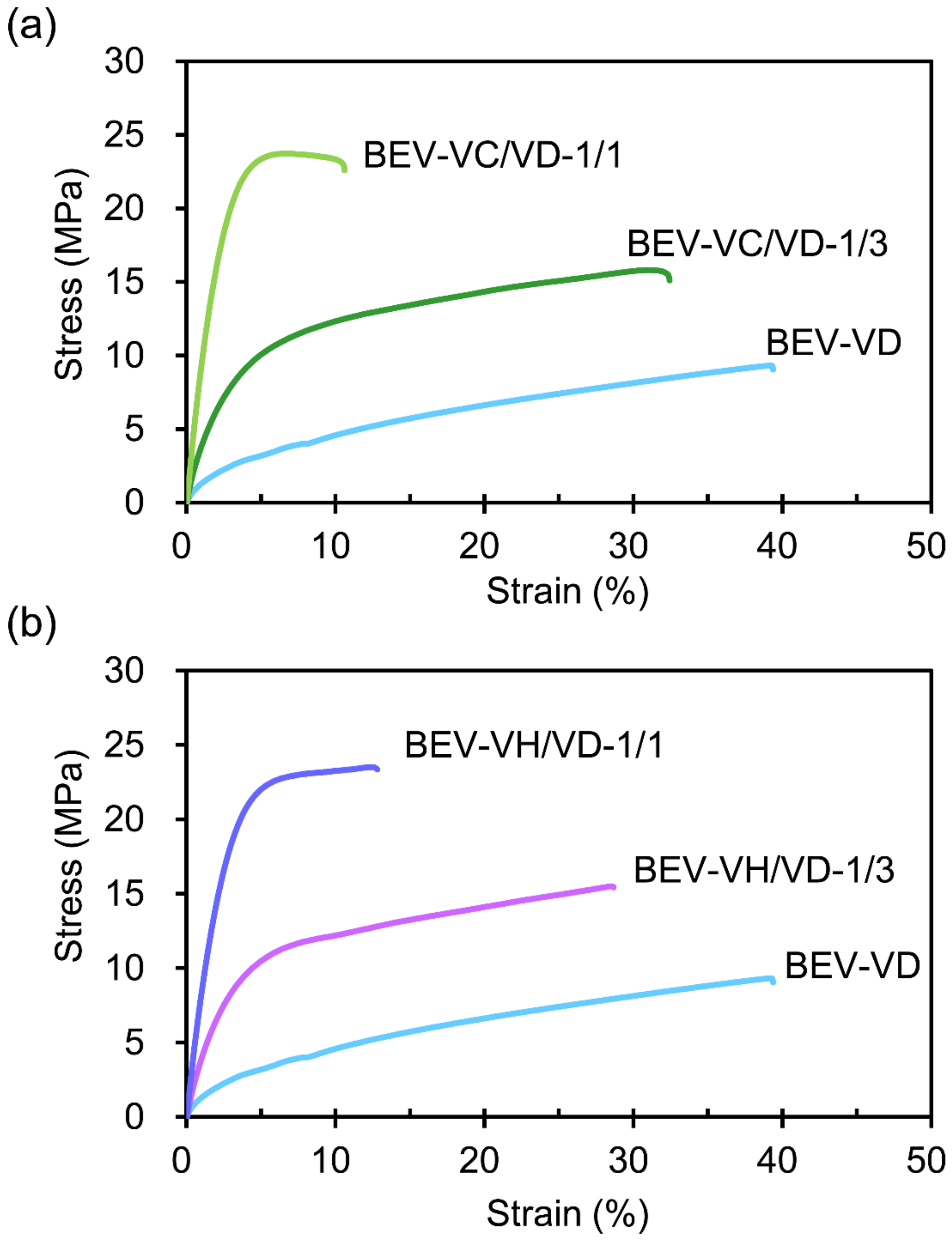

Figure S4. Tensile stress-strain curves of (a) BEV-VC/VD-1/1, BEV-VC/VD-1/3, and BEV-VD, and (b) BEV-VH/VD-1/1, BEV-VH/VD-1/3, and BEV-VD.

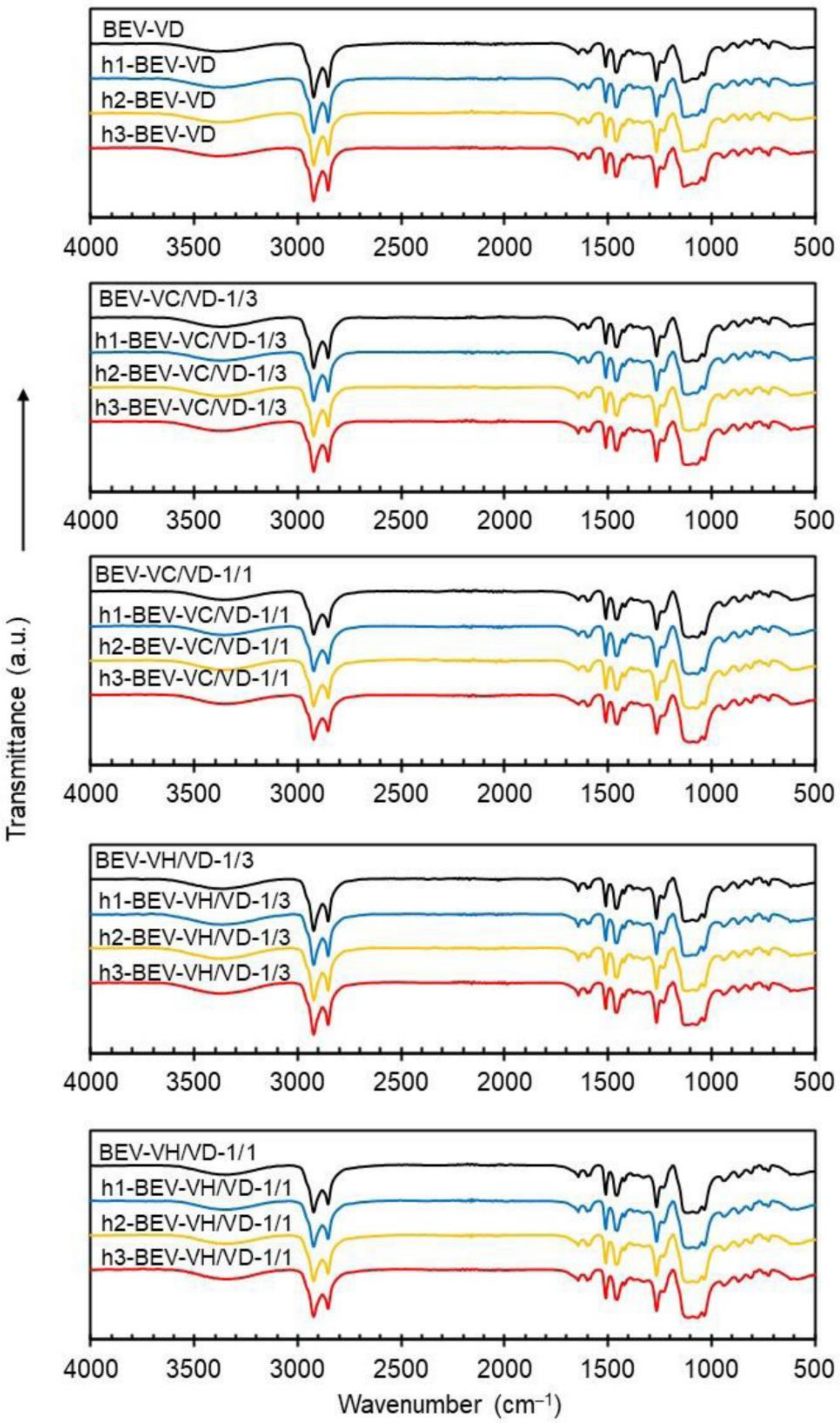

Figure S5. FT-IR spectra of h1-, h2-, and h3-BEV-VD, BEV-VC/VD, and BEV-VH/VD films.

Table S1. Tensile moduli, tensile strengths and elongation at breaks of original-, h1-, h2-, and h3-BEV-VD, BEV-VC/VD, and BEV-VH/VD films.

Sample	Tensile modulus (MPa)	Tensile strength (MPa)	Elongation at break (%)	η_σ (%)
original-BEV-VD	191 ± 24	9.29 ± 0.15	41.6 ± 4.46	-
h1-BEV-VD	187 ± 37	6.93 ± 0.82	26.9 ± 0.72	74.7 ± 8.9
h2-BEV-VD	155 ± 7	3.65 ± 0.38	10.4 ± 1.93	39.4 ± 4.1
h3-BEV-VD	171 ± 7	3.05 ± 0.11	6.15 ± 1.37	32.8 ± 1.3
original-BEV-VC/VD-1/3	545 ± 42	16.1 ± 0.97	32.8 ± 2.10	-
h1-BEV-VC/VD-1/3	465 ± 94	12.5 ± 0.27	15.1 ± 3.38	77.8 ± 5.0
h2-BEV-VC/VD-1/3	418 ± 102	9.84 ± 1.04	6.36 ± 0.38	61.2 ± 7.5
h3-BEV-VC/VD-1/3	489 ± 88	7.78 ± 0.17	2.85 ± 0.63	48.4 ± 3.1
original-BEV-VC/VD-1/1	1067 ± 26	23.3 ± 0.87	9.33 ± 1.82	-
h1-BEV-VC/VD-1/1	1036 ± 46	17.9 ± 0.59	2.44 ± 0.21	76.8 ± 3.9
h2-BEV-VC/VD-1/1	1147 ± 25	10.8 ± 0.66	1.10 ± 0.08	46.3 ± 3.3
h3-BEV-VC/VD-1/1	1124 ± 40	7.83 ± 1.00	0.78 ± 0.13	33.6 ± 4.5
original-BEV-VH/VD-1/3	489 ± 54	16.3 ± 0.89	30.7 ± 3.77	-
h1-BEV-VH/VD-1/3	562 ± 73	12.3 ± 0.86	7.48 ± 2.43	75.5 ± 6.7
h2-BEV-VH/VD-1/3	531 ± 33	9.15 ± 0.61	3.49 ± 0.57	56.0 ± 4.8
h3-BEV-VH/VD-1/3	515 ± 26	6.75 ± 0.24	2.10 ± 0.13	41.4 ± 2.7
original-BEV-VH/VD-1/1	973 ± 64	23.6 ± 0.78	13.0 ± 2.72	-
h1-BEV-VH/VD-1/1	852 ± 124	18.0 ± 1.47	3.46 ± 0.73	76.3 ± 6.7
h2-BEV-VH/VD-1/1	861 ± 47	10.9 ± 1.08	1.59 ± 0.18	46.3 ± 4.8
h3-BEV-VH/VD-1/1	866 ± 44	7.46 ± 0.25	1.02 ± 0.07	31.6 ± 1.5
